# Ets-1 global gene expression profile reveals associations with metabolism and oxidative stress in ovarian and breast cancers

**DOI:** 10.1186/2049-3002-1-17

**Published:** 2013-07-25

**Authors:** Meghan L Verschoor, Chris P Verschoor, Gurmit Singh

**Affiliations:** 1 Department of Medical Science, McMaster University, 1280 Main Street W, Hamilton, Ontario L8N 3Z5, Canada; 2 Department of Pathology and Molecular Medicine, McMaster University, 1280 Main Street W, Hamilton, Ontario L8N 3Z5, Canada; 3 Juravinski Cancer Centre, 699 Concession Street, Hamilton, Ontario L8V 5C2, Canada

**Keywords:** Breast cancer, Ets-1, Metabolism, Ovarian cancer, Oxidative stress

## Abstract

**Background:**

The Ets-1 proto-oncogene is frequently upregulated in cancer cells, with known involvement in cancer angiogenesis, metastasis, and more recently energy metabolism. In this study we have performed various bioinformatic analyses on existing microarray data to further clarify the role of Ets-1 in ovarian cancer, and validated these results with functional assays.

**Methods:**

Functional pathway analyses were conducted on existing microarray data comparing 2008 and 2008-Ets1 ovarian cancer cells. Methods included over-representation analysis, functional class scoring and pathway topology, and network representations were visualized in Cytoscape. Oxidative stress regulation was examined in ovarian cancer cells by measuring protein expression and enzyme activity of glutathione peroxidases, as well as intracellular reactive oxygen species using dichlorofluorescin fluorescence. A stable Ets-1 knockdown MDA-MB-231 cell line was created using short hairpin RNA, and glycolytic dependence of these cells was measured following treatment with 2-deoxy-D-glucose and Hoechst nuclear staining to determine cell number. High-resolution respirometry was performed to measure changes in basal oxygen flux between MDA-MB-231 cells and MDA-Ets1KD variants.

**Results:**

Enrichments in oxidoreductase activity and various metabolic pathways were observed upon integration of the different analyses, suggesting that Ets-1 is important in their regulation. As oxidative stress is closely associated with these pathways, we functionally validated our observations by showing that Ets-1 overexpression resulted in decreased reactive oxygen species with increased glutathione peroxidase expression and activity, thereby regulating cellular oxidative stress. To extend our findings to another cancer type, we developed an Ets-1 knockdown breast cancer cell model, which displayed decreased glycolytic dependence and increased oxygen consumption following Ets-1 knockdown confirming our earlier findings.

**Conclusions:**

Collectively, this study confirms the important role of Ets-1 in the regulation of cancer energy metabolism in ovarian and breast cancers. Furthermore, Ets-1 is a key regulator of oxidative stress in ovarian cancer cells by mediating alterations in glutathione antioxidant capacity.

## Background

Ets-1 is a member of the E-26 (Ets) family of transcription factors, and was first characterized as a proto-oncogene of the retroviral *v-ets* oncogene in avian leukemia retrovirus E26 [1]. This family of transcription factors currently comprises 28 members, many of which are known to be elevated in various cancers [2,3] including Ets-2 [4-9], Friend leukemia integration 1 [10], Ets-related gene [4], Polyomavirus enhancer activator 3 homolog [11,12], Ets-related molecule [11], Prostate epithelium-specific Ets transcription factor [13] and E74-like factor-3 [11]. All known Ets family members contain a core double-stranded DNA binding element that recognizes the consensus sequence GGAA/T [14,15]. Because the Ets binding element is simple and generic, there is significant functional redundancy among Ets factors, allowing for complex transcriptional networks depending on which factors are bound to a specific promoter. The diverse functional roles of these factors include differentiation, proliferation, apoptosis, angiogenesis, malignant transformation and metastasis, which are all processes relevant to the study of cancer.

High levels of Ets-1 expression are observed in a wide variety of cancer types including those of the breast, prostate and ovary; this suggests that the association between Ets-1 expression and tumor progression is a generalized phenomena [16]. Ets-1 upregulation appears to associate specifically with more advanced, invasive tumors in breast and ovarian carcinomas [17-22], and is positively correlated with the enhanced metastatic potential of numerous cancers [17,23-26]. Indeed, there are many well-established target genes for Ets-1 that are closely linked to cancer progression, particularly mediators of extracellular matrix degradation, cancer cell migration and angiogenesis [16,25,27-31]. Thus, the consequences of Ets-1 overexpression are particularly relevant to the study of ovarian cancer as this type of malignancy is very difficult to detect, and is most commonly diagnosed at advanced stages of disease progression that include metastases. Comparing the transcriptional programs of cancer cells that express low levels of Ets-1 protein to those that express Ets-1 protein in abundance will create a gene expression profile illustrating some of the key differences between invasive and non-invasive ovarian cancer cells.

Recently, our laboratory showed the importance of Ets-1 as a regulator of cellular metabolism in ovarian cancer cells, where Ets-1 overexpression resulted in increased glycolysis while suppressing oxidative phosphorylation, a phenomena known as the Warburg effect [32]. The objective of the present study was to examine the functional interactions of the potential downstream targets of Ets-1 identified in the microarray analysis from our previous work. In our previous study, we used a stable Ets-1 overexpression model in 2008 ovarian cancer cells to conduct whole genome microarray analysis, which we have more comprehensively examined here to further clarify the role of Ets-1 in ovarian tumorigenesis. We have utilized three different approaches of bioinformatic pathway analysis, and compared them to identify the pathway associations that are common to each method in order to delineate the most important pathways represented following Ets-1 overexpression. The findings from our pathway-based network analyses illustrate the importance of Ets-1 expression in cancer-associated metabolic regulation in ovarian cancer.

The most novel finding among other commonly enriched functional pathways we identified was likely that of pathways involving the regulation of cellular redox status. To provide some validation for this finding, we examined the protein expression of elevated targets involved in the regulation of cellular redox status, and measured intracellular reactive oxygen species (ROS) production in ovarian cancer cells overexpressing Ets-1. Additionally, to investigate the ability of our findings to extend to other types of human cancer, we developed an Ets-1 knockdown model in MDA-MB-231 breast cancer cells, as aggressive breast cancer is frequently associated with the overexpression of Ets-1 [17,21,22,33]. Because metabolic pathways were the most common pathway association with changes to Ets-1, and because we have previously established an association between energy metabolism and Ets-1 in ovarian cancer cells, we used this model to examine the significance of Ets-1 knockdown on glycolysis and oxidative phosphorylation. We observed that Ets-1 regulation of cancer-specific metabolic changes is a phenomenon that also exists in breast cancer cells, thus complementing our previous work and suggesting that Ets-1 regulation of metabolism may be a generalized phenomenon.

## Methods

### Cell culture

The human ovarian carcinoma cell line 2008 was kindly provided by Dr Paul Andrews (Georgetown University, Rockville, MD, US) [34]. MDA-MB-231 human mammary epithelial adenocarcinoma cells were obtained from the American Type Culture Collection (ATCC# HTB-26). The 2008 cells were maintained in Roswell Park Memorial Institute 1640 medium supplemented with 10% fetal bovine serum and 2% penicillin/streptomycin. MDA-MB-231 cells were maintained in Dulbecco’s modified Eagle’s medium supplemented with 10% fetal bovine serum and 2% penicillin/streptomycin. Stable cell lines 2008-Ets1 and MDA-Ets1KD were maintained in growth medium as described with the addition of 200 ng/ml selective antibiotic. All cells were kept at 37°C in a humidified atmosphere of 5% CO_2_. Media and supplements were purchased from Invitrogen Life Technologies (Burlington, ONT, Canada), and fetal bovine serum from Fisher Scientific (Ottawa, ONT, Canada). All reagents were purchased from Sigma (Oakville, ONT, Canada).

### Microarray pathway analysis

Ontological analysis was performed using the online software DAVID [35]. Gene lists composed of probe sets showing absolute linear fold-change differences ≥2 and q-values ≤0.05 were compared against the HuGene-1_0-st-v1 background list supplied by DAVID. Functional annotation charts were produced for ontological categories of interest comprising at least 10 genes, and functional annotation clustering was performed at high classification stringency. Ranked gene set enrichment analysis (GSEA) was performed to identify significantly enriched gene sets between 2008 and 2008-Ets1 gene expression profiles as described previously [36,37].

Complex network analysis and visualization was performed using Cytoscape, an open source bioinformatics software package [38]. The Network analyzer plugin was used to perform statistical calculations and visualizations of topological parameters and centrality measures for the biological networks generated from the various analyses [39]. Normalized gene expression data from the microarray was used to create a large, comprehensive network using Reactome Function Interaction (FI) using a size of 10 and an average correlation minimum of 0.75 [40]. This plugin utilizes the Reactome FI network of human protein interactions to create a specific sub-network based on the differentially regulated genes in the microarray. The Enrichment Map plugin [41] was used to generate networks derived from the GSEA results, and the MCODE plugin [42] was used to further cluster the GSEA enrichment map.

### Protein isolation and western blot analysis

Whole cell lysates were collected, 30 μg of protein was separated by 10% SDS-PAGE electrophoresis, transferred to polyvinylidene difluoride membrane, and blocked for 1 h in 5% skim milk Tris-buffered saline with Tween. Membranes were incubated overnight with antibody reactive to Ets-1 (Abcam, Boston, MA, US), glutathione peroxidases 1 and 2 (GPX1 and 2; Abcam) or Actin (Cell Signaling, Danvers, MA, US) in 0.5% Tris-buffered saline with Tween. Following primary antibody incubation, membranes were washed and incubated for 2 h with horseradish peroxidase-linked anti-mouse or anti-rabbit immunoglobulin G secondary antibody (Cell Signaling). Proteins were detected by ECL chemiluminescence reagent (Amersham Biosciences, Baie D’Urfe, QC, Canada), and exposed to film. Densitometry analysis was performed using ImageJ software developed by Wayne Rasband, National Institutes of Health, Bethesda, MD, USA [43].

### Intracellular reactive oxygen species and glutathione peroxidase activity assays

Intracellular ROS levels were measured using CM_2_-H_2_DCFDA reagent (Invitrogen), which is cleaved once inside the cell allowing the DCF dye to bind to ROS species, resulting in fluorescence. Cells were plated in 96-well plates and grown to 70% to 90% confluency in phenol red-free medium. CM_2_-H_2_DCFDA reagent was reconstituted in dimethyl sulfoxide, and 10 μM was added to each experimental well using phenol red-free medium containing 10% fetal bovine serum. Following a 30 min incubation to allow the dye to load into cells, plates were washed twice with phosphate-buffered saline, and allowed to recover in phenol red-free medium for 10 minutes. Plates were then treated with 250 μM H_2_O_2_ and read in a Cytofluor fluorescent plate reader at 485 nm excitation and 530 nm emission for 1 h. Plates were then stained with crystal violet, dried overnight, solubilized with SDS, and read at 570 nm. Arbitrary fluorescent values were normalized to crystal violet absorbance values, and reported as arbitrary fluorescent units (AFU). Glutathione peroxidase enzyme activity was measured using the GPX activity kit from Enzo Life Sciences (Farmingdale, NY, USA) according to the manufacturer instructions.

### Ets-1 knockdown breast cancer model

MDA-MB-231 cells were plated in 6-well plates at 1.5 × 10^5^ cells per well, incubated for 24 h, then transfected with 1 μg of the pSM2 retroviral vector (Open Biosystems, Huntsville, AL, US) containing a pre-designed short hairpin RNA sequence targeting Ets-1 (SH2588-A-5) or a non-silencing control vector (RHS1703) using Arrest-In^to^ (Open Biosystems). Plates were exposed to selection medium containing 2 μg/ml Puromycin over 3 weeks, and surviving clones were isolated, expanded and tested for Ets-1 protein expression via western blotting. The clone with the lowest Ets-1 protein expression was expanded and will be referred to as MDA-Ets1KD cells in this study.

### RNA isolation and quantitative real-time PCR

Total RNA was isolated using Trizol reagent as indicated by the manufacturer (Invitrogen). RNA samples were DNase treated using Turbo DNA-free™ as per the manufacturer’s directions (Invitrogen), and 3 μg of total cellular RNA was reverse-transcribed using the Superscript III First Strand Synthesis System (Invitrogen). Quantitative real-time PCR was conducted using Platinum SYBR Green qPCR SuperMix UDG (Invitrogen) with primers sequences listed in Additional file 1. All target gene expression was normalized to the pooled gene expression values of β-actin, B-2 macroglobulin, glyceraldehyde-3-phosphate dehydrogenase and RNA polymerase II as housekeeping controls. Data were normalized and efficiency corrected using the ΔΔCt method of relative quantification, where statistical significance and standard error were determined from ΔCt values.

### Measurement of glycolytic dependency

The glycolytic dependency assays were performed as described previously [32]. Briefly, cells were treated with 2-deoxy-D-glucose (2-DG), which was administered at concentrations ranging from 1 to 7.5 mM over 96 h, at which time a Hoechst DNA content assay (Invitrogen) was performed. Cell number was standardized to fluorescence for each cell type by comparison with a standard curve of known cell numbers.

### Oxygen consumption assay

The oxygen consumption assay was performed as described previously [32]. Briefly, harvested cells were added to the oxygraph chambers containing 1.8 mL of KCl medium and the total O_2_ concentration and flux was recorded at one second intervals throughout the duration of the experiment. Once the oxygen concentration stabilized, cells were permeabilized with digitonin and treated with various respiratory substrates and inhibitors. OROBOROS software was used for data acquisition and analysis (OROBOROS Instruments GmbH, Innsbruck, Austria).

### Statistical analysis

Data is presented as the mean ± SD from at least three independent experiments. Statistically significant differences between sample groups were determined using a Student’s t-test or analysis of variance where applicable, with a *P*-value ≤0.05 considered to be statistically significant.

## Results

### Global gene expression analysis

We previously performed comparative microarray analysis of 2008 and 2008-Ets1 ovarian cancer cells [32,44], which revealed a large amount of differentially regulated genes. Subsequent real-time qRT-PCR validation was examined, and gene expression changes greater than 1.5-fold were deemed valid and included in this study. The complete raw data is available via the GEO database under accession number [GEO:GSE21129].

**Figure 1:**
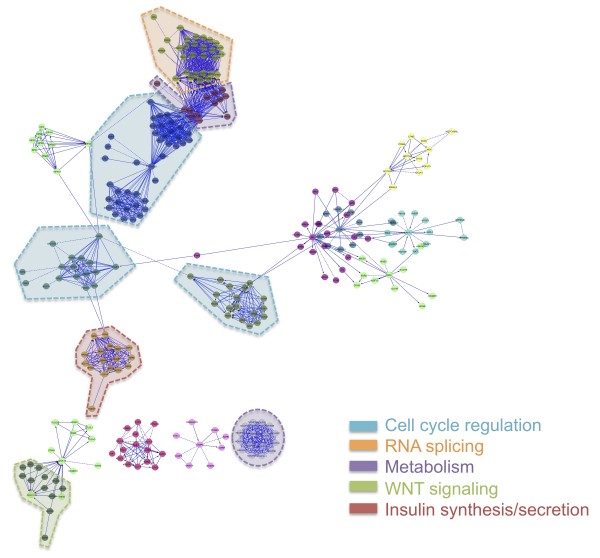
**Global functional interaction network.** Reactome FI was used to create a functional interaction network, which is divided into modules as defined by node coloring, and further delineated by functional pathway enrichments into outlined, color-coded groups. Major associations among the curated and predicted interactions included cell cycle regulatory, RNA slicing, metabolism, WNT signaling, and insulin-related pathways (FDR <0.01). Network was generated in Cytoscape.

Reactome FI was used to create a global human interaction network for the differentially regulated genes in our microarray data set (Figure 1). This plugin was specifically created to find network patterns pertaining to cancer, by building a network of highly interacting gene groups, referred to as modules. To decrease the complexity of the network generated, the module size was set to a minimum of 10 interacting genes, average correlation set to 0.75, and false discovery rate (FDR) set to 0.25 [40]. The resulting network contained 275 nodes of curated and predicted protein interactions clustered into 16 functional modules as delineated by different node coloring (Figure 1). Functional pathway enrichment analysis (FDR <0.01) was performed on the large network to identify the major pathways and ontological terms associated with each module of interacting genes. The module functions with the highest gene set ratio, and thereby the most important pathways annotated, included cell cycle regulation, RNA splicing, wingless-type (WNT) signaling and metabolic pathways, as well as insulin synthesis and secretion (identified by colored outlines in Figure 1). The gene set ratio is a measure of the number of enriched genes present in a data set compared to the total number of genes within the curated gene set, and thus a good indicator of the relative importance of each pathway within the data set. The individual genes contained in the most important outlined modules are detailed in Additional file 2.

### Gene ontology

Functional annotation clustering of the differentially regulated genes was completed using DAVID, an online database of functional annotation tools. This software identifies which annotation groups are enriched in a list of differentially expressed genes by grouping functionally similar terms associated with these genes into annotation clusters. Meaningful functional relationships can then be inferred from the functional categories that are significantly over-represented within the list of enriched genes. Under the highest classification stringency, eight annotation clusters were identified as significantly enriched in our microarray data set (Table 1). Clusters relevant to our functional pathway enrichment analysis (Figure 1) included oxidoreductase activity relating to fatty acid desaturation (cluster 1), unsaturated fatty acid metabolism (cluster 2), D-Aspartate:2-oxoglutarate aminotransferase activity (cluster 3), and dihydrodiol dehydrogenase activity (cluster 4). We have reported the *P*-value and fold enrichment for each annotation cluster. The *P*-value represents the threshold of EASE Score, which is a modified Fisher exact *P*-value used for gene-enrichment analysis to determine the significance of the gene set enrichment. The measure of fold enrichment represents a ratio of the proportion of input genes present within a gene set compared to the number of genes that term represents in the human genome background data set [35]. Thus, a low *P*-value combined with a high fold enrichment together indicate a high magnitude of enrichment for that particular ontology term. Our findings indicated that the best enrichments relating to Ets-1 overexpression included fatty acid desaturation (annotation clusters 1 and 2) and dihydrodiol dehydrogenase activity (annotation cluster 4).

**Table 1:** Functional annotation clustering of genes associated with Ets-1 overexpression

**Annotation cluster**	**Index**	**Term**	***P***	**Fold enrichment**
1	INTERPRO	IPR010257:Fatty acid desaturase, type 1, N-terminal	0.00	15.75
	GOTERM_MF_ALL	GO:0016717~Oxidoreductase activity (O or H_2_O as acceptor)	0.00	13.63
	INTERPRO	IPR005804:Fatty acid desaturase, type 1	0.00	13.13
2	PANTHER_FAMILY	PTHR19353:Fatty acid desaturase 2	0.001	20.40
	INTERPRO	IPR012171:Fatty acid/sphingolipid desaturase	0.001	19.69
	PIR_SUPERFAMILY	PIRSF015921:Fatty acid desaturase/sphingolipid desaturase	0.001	19.22
	GOTERM_BP_ALL	GO:0006636~Unsaturated fatty acid biosynthetic process	0.001	14.89
	GOTERM_BP_ALL	GO:0033559~Unsaturated fatty acid metabolic process	0.001	14.89
3	KEGG_REACTION	R05053:D-aspartate + D-4-hydroxy-2-oxoglutarate <=> Oxaloacetate + L-erythro-4-hydroxyglutamate	0.001	2.78
	KEGG_COMPOUND	C05947:L-erythro-4-hydroxyglutamate	0.001	2.76
	KEGG_COMPOUND	C05946:D-4-hydroxy-2-oxoglutarate	0.001	2.76
	KEGG_COMPOUND	C00036:Oxaloacetate	0.001	2.76
	KEGG_COMPOUND	C00402:D-aspartate	0.001	2.76
4	GOTERM_MF_ALL	GO:0047115~trans-1,2-dihydrobenzene-1,2-diol dehydrogenase activity	0.001	20.44
	EC_NUMBER	1.1.1.213	0.001	16.92
	EC_NUMBER	14.3.1.20	0.001	16.92
5	PIR_SUPERFAMILY	PIRSF001191:Matrix metalloproteinase, stromelysin type	0.01	5.34
	INTERPRO	IPR002477:Peptidoglycan binding-like	0.02	4.92
	PANTHER_FAMILY	PTHR10201:Matrix metalloproteinase	0.02	4.43
	INTERPRO	IPR000585:Hemopexin	0.03	4.10
	INTERPRO	IPR006026:Peptidase, metallopeptidases	0.04	3.79
	GOTERM_BP_ALL	GO:0000270~Peptidoglycan metabolic process	0.05	3.54
6	INTERPRO	IPR001314:Peptidase S1A, chymotrypsin	0.02	2.28
	INTERPRO	IPR001254:Peptidase S1 and S6, chymotrypsin/Hap	0.04	2.10
	PANTHER_FAMILY	PTHR19355:Serine protease-related	0.04	2.10
7	INTERPRO	IPR001039:MHC class I, alpha chain, alpha1 and alpha 2	0.02	4.69
	PANTHER_FAMILY	PTHR16675:MHC class 1-related	0.03	4.08
	INTERPRO	IPR011161:MHC classi-like antigen recognition	0.04	9.94
	GOTERM_CC_ALL	GO:0042612~MHC class I protein complex	0.04	3.91
8	INTERPRO	IPR010579:MHC class I, alpha chain, C-terminal	0.01	14.77
	PFAM	PF06623:MHC_I_C	0.01	14.63

### Gene set enrichment analysis

**Figure 2:**
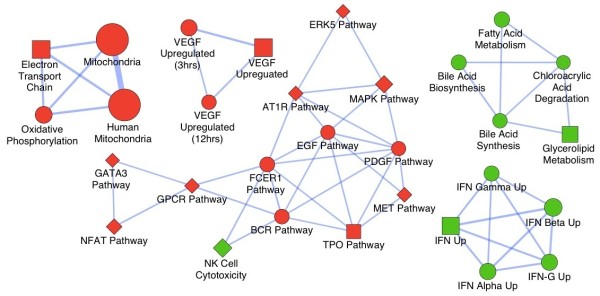
**Enrichment map of gene set enrichment analysis.** The map shows enriched gene sets in 2008 versus 2008-Ets1 ovarian cancer cells clustered by MCODE to generate sub-networks of the interrelated gene sets. Red nodes indicate enrichment (upregulation) in 2008 cells, green nodes represent enrichment (upregulation) in 2008-Ets1 cells. Node size is representative of the number of enriched genes in the gene set. The largest cluster includes the signaling pathways of ERK5, MAPK, EGF, PDGF, MET and GPCR. Notably, clusters with gene sets involved in mitochondrial metabolism and fatty acid metabolic processes were also identified. Network was generated in Cytoscape.

To examine the biological pathways enriched in the data set in an unbiased, systematic method, GSEA was performed [36]. This software examines gene expression data at the level of gene sets, which are based on existing biological pathway or co-expression data from published research within the Molecular Signature Database. GSEA results were also applied to Enrichment Map in Cytoscape to generate a large network of enriched gene sets, then the large network was clustered using MCODE to generate five sub-networks of interrelated gene sets (Figure 2). The largest cluster included several signaling pathways, most notably extracellular-signal-regulated kinase 5 (ERK5), mitogen-activated protein kinase (MAPK), epidermal growth factor (EGF), platelet-derived growth factor (PDGF), MET proto-oncogene and G protein-coupled receptor (GPCR) pathways. Relevant to our other analyses, clusters containing gene sets involved in mitochondrial metabolism and fatty acid metabolic processes were also identified (gene lists provided in Additional file 3). More detailed description of these gene sets can be found in the MsigDB database [36].

### Comparison of bioinformatic data

**Figure 3:**
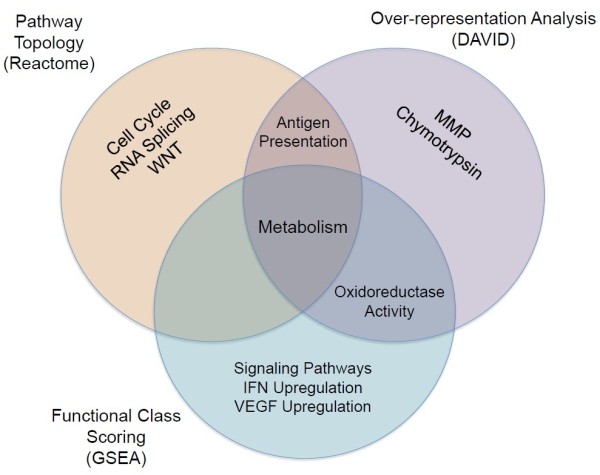
**Integrating various bioinformatic analyses.** Venn diagram representing the overlapping enrichments from the various bioinformatic pathway analyses (Figures 1 and 2, Table 1) employed on the microarray expression data. The functional interaction network and ontological analyses both included enrichments in antigen presentation, and the ontological analysis shared oxidoreductase activity enrichment with the gene set enrichment analysis. All three analyses found enrichments in various metabolic pathways.

Pathway analysis of microarray data is a powerful tool for the identification of the underlying biological significance of a large gene expression lists, allowing for improved understanding of high-throughput data. There exist three distinct methods for conducting pathway analyses, including over-representation analysis, functional class scoring and pathway topology analysis. Each approach generates a list of significantly enriched pathways present within the input gene list, however there are limitations and advantages to each of these methods as reviewed by Khatri *et al.*[45]. For this study, we chose to analyze our gene expression data using each of these approaches, and then compared the results to determine the most important pathway associations present following Ets-1 overexpression in 2008 ovarian cancer cells. Oxidoreductase activity was a common pathway association between DAVID (over-representation analysis) and GSEA (functional class scoring) methods, whereas antigen presentation was enriched in both GSEA (functional class scoring) and Reactome (pathway topology analysis) analyses (Figure 3). Interestingly, metabolic pathway associations were found using all analysis methods, suggesting that this observation is the most significant finding within our Ets-1 overexpression model of ovarian cancer.

### Ets-1 overexpression affects oxidative stress in cancer cells

**Figure 4:**
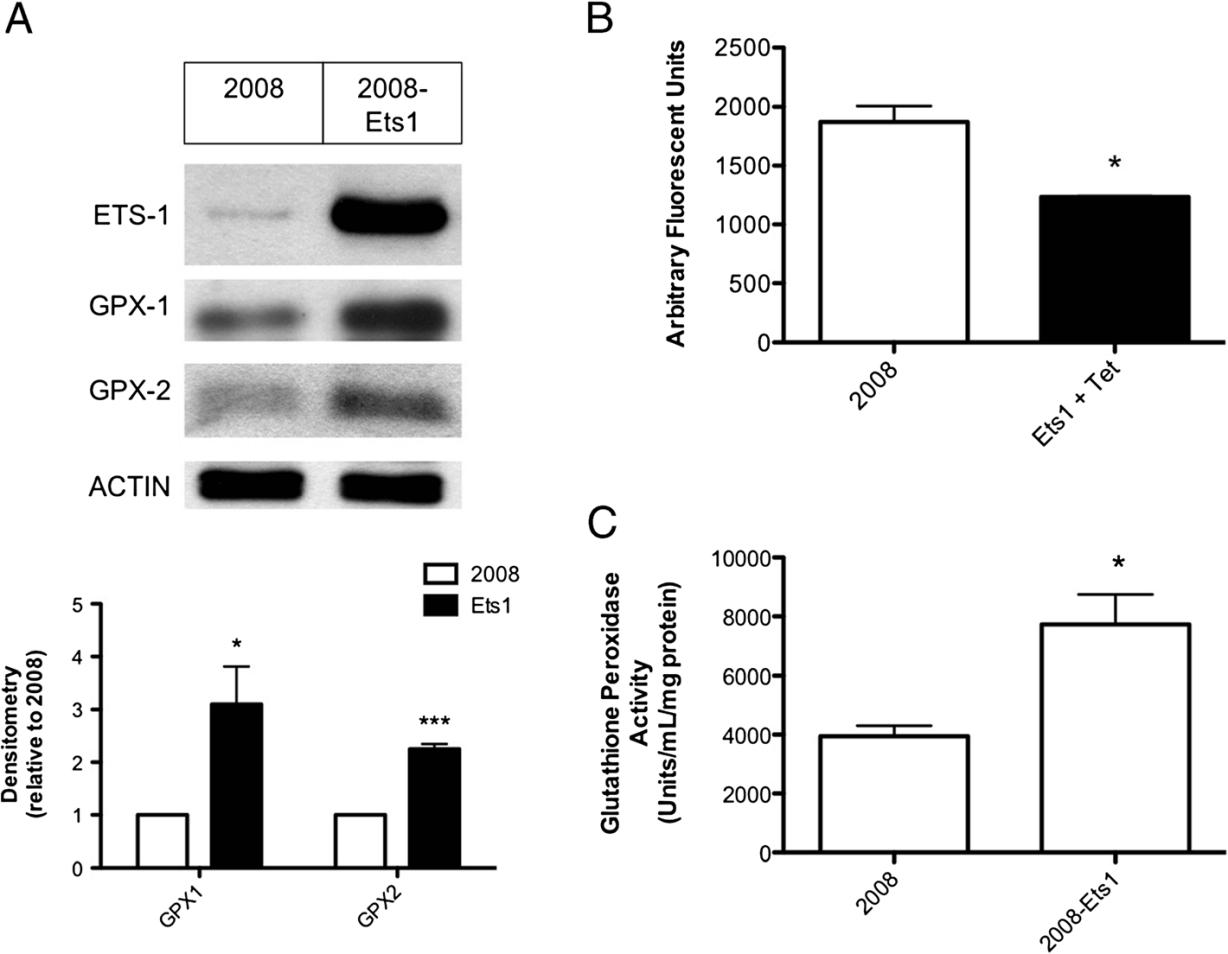
**Ets-1 regulated oxidative stress in ovarian cancer cells. (A)** The protein expression of Ets1, GPX-1 and GPX-2 were examined via western blot, and normalized to Actin expression by densitometry analysis. Ets-1 overexpressing cells show 3.10-fold and 2.25-fold inductions in GPX-1 and GPX-2 protein levels, respectively. **(B)** Intracellular ROS levels were measured using the fluorescent CM_2_-H_2_DCFDA reagent in ovarian cancer cells. 2008-Ets1 cells contained lower ROS levels than 2008 cells (1233.99 AFU and 1872.73 AFU, respectively). **(C)** The activity of glutathione peroxidase enzymes was measured using a colorimetric assay, where Ets-1 overexpressing cells were observed to have significantly higher activity than parental cells (7725.66 U/mL/mg and 3944.22 U/mL/mg, respectively).

Because enrichments in various metabolic pathways and oxidoreductase activity were observed in each of the bioinformatic analyses we examined (Figure 3), we investigated the association between oxidative stress and Ets-1 expression. The upregulation of GPX-1 and GPX-2 was validated via western blot, where the protein expression of both factors was increased in response to Ets-1 induction by 3.10-fold and 2.25-fold respectively (Figure 4A). Functionally, intracellular ROS levels were significantly lower in 2008-Ets1 cells (1233.99 AFU) compared to 2008 cells (1872.73 AFU) (Figure 4B). The activity of GPX enzymes was significantly higher in 2008-Ets1 cells (7725.66 U/mL/mg) than in 2008 cells (3944.22 U/mL/mg) (Figure 4C).

### Breast cancer cell metabolism is regulated by Ets-1

**Figure 5:**
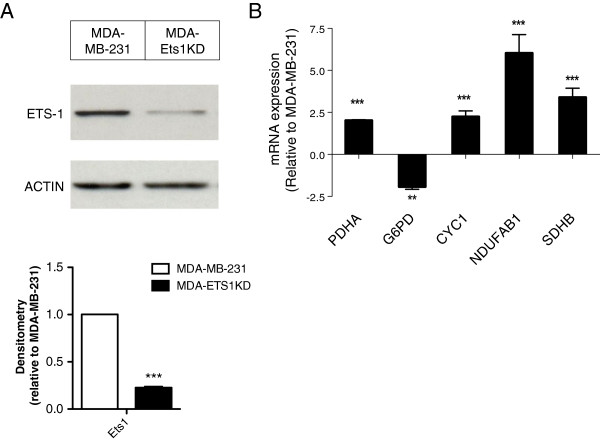
**Breast cancer cell model of Ets-1 expression knockdown.** MDA-MB-231 breast cancer cells were stably depleted of Ets-1 expression via targeted shRNA knockdown. **(A)** The Ets-1 knockdown cell line MDA-Ets1KD expresses Ets-1 protein at 22.7% of parental protein levels in MDA-MB-231 cells. **(B)** Real-time qRT-PCR of the breast cancer Ets-1 expression model. The gene expression of *PDHA*, *CYC1*, *NDUFAB1* and *SDHB* were increased in MDA-Ets1KD cells, whereas the expression of *G6PD* was downregulated.

As we have previously shown that Ets-1 regulates energy metabolism in ovarian cancer cells, for this study we endeavored to extend those findings to breast cancer cells. The Ets-1 knockdown cell line MDA-Ets1KD was generated, because the aggressive MDA-MB-231 breast cancer cell line expresses Ets-1 in abundance, where Ets-1 expression is decreased to 22.7% of parental levels (Figure 5A). To determine whether this cell model expressed the same differences in metabolic genes as we observed in our previous microarray analysis [32], we performed real-time qRT-PCR on the breast cancer cell model. The gene expression of *PDHA*, *CYC1*, *NDUFAB1* and *SDHB* were increased in MDA-Ets1KD cells, whereas the expression of *G6PD* was downregulated (Figure 5B). The inverse relationship observed between the expression levels of these genes in our ovarian and breast cancer cell models suggest that Ets-1 regulates the expression of these factors in a similar manner in breast cancer cells.

**Figure 6:**
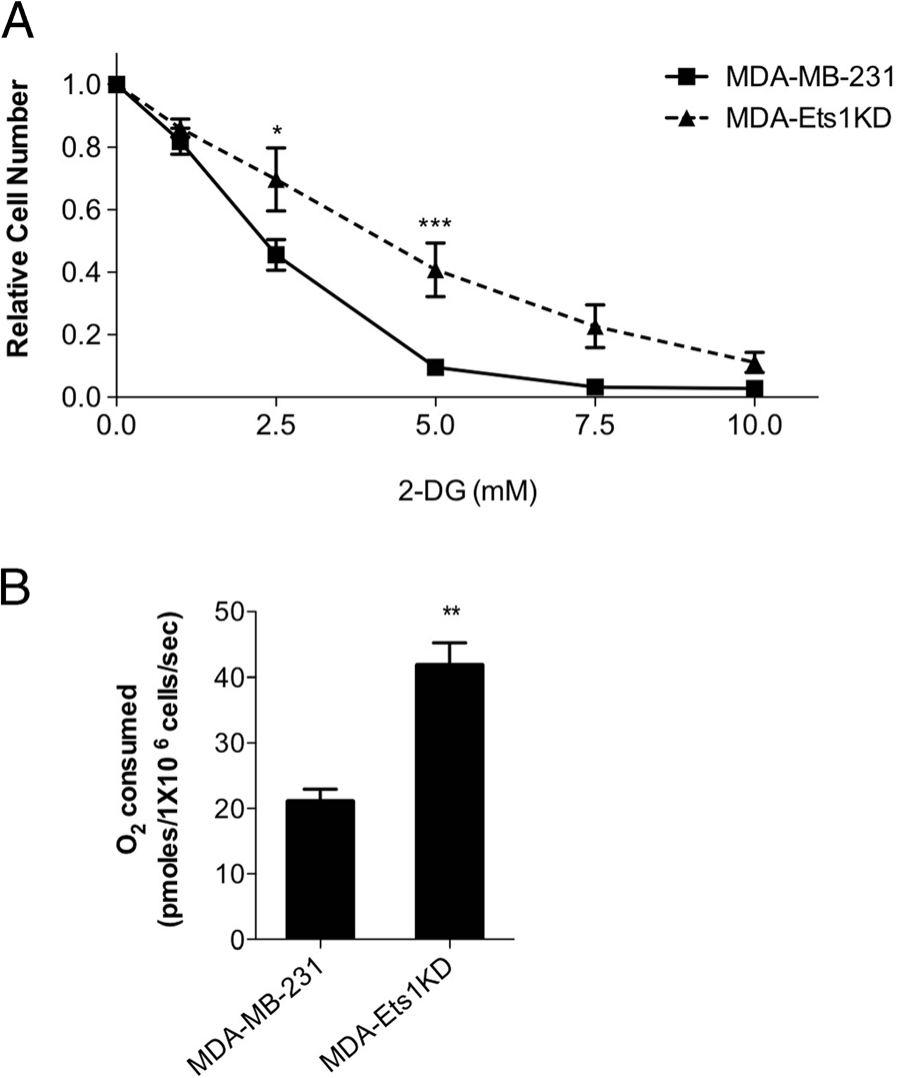
**Effect of Ets-1 knockdown on breast cancer cell metabolism. (A)** MDA-MB-231 and MDA-Ets1KD cells were treated with various amounts of the glycolytic inhibitor 2-DG, and representative growth curves were generated for each cell line. The 2-DG IC_50_ was greater in MDA-Ets1KD cells, with an IC_50_ of 3.94 mM compared to an IC_50_ of 2.03 mM for MDA-MB-231 cells. **(B)** Basal oxygen consumption was measured using high-resolution respirometry, and MDA-Est1KD cells were observed to consume significantly more oxygen (41.80 pmol/10^6^ cells/s) than parental MDA-MB-231 cells (21.07 pmol/10^6^ cells/s).

To examine the functional consequences of decreased Ets-1 expression in breast cancer cells, we examined glycolytic dependence and oxygen consumption in MDA-Ets1KD cells. Cells were treated with various amounts of the glycolytic inhibitor 2-DG, and representative growth curves were generated for each cell line. When inhibited with 2-DG, the growth of MDA-Ets1KD cells was decreased to a lesser extent than that of MDA-MB-231 cells (Figure 6A). The dose at which 50% of cells had stopped proliferating, or 2-DG IC_50_, was greater in MDA-Ets1KD cells with an IC_50_ of 3.94 mM compared to an IC_50_ of 2.03 mM for MDA-MB-231 cells. Basal oxygen consumption was measured using high-resolution respirometry, and MDA-Est1KD cells were observed to consume significantly more oxygen (41.80 pmol/10^6^ cells/s) than parental MDA-MB-231 cells (21.07 pmol/10^6^ cells/s) (Figure 6B).

## Discussion

Our analysis shows that the expression of Ets-1 in cancer cells results in a transcriptional program that confers enhanced cancer progression and development through the alteration of metabolism and redox status. Ets-1 is widely expressed by tumor cells, endothelial cells and tumor-associated fibroblasts, where it is known to contribute to tumor angiogenesis and cancer cell invasion [20,29,30,46-64]. Ets transcription factors are increasingly associated with such interactions between tumor and stromal cells, particularly in the context of extracellular matrix remodeling. Numerous Ets proteins are aberrantly expressed in both tumor and stromal cells, resulting in the overexpression of tumor-promoting factors such as matrix metalloproteinase (MMP)-1, MMP-3, MMP-9, urokinase plasminogen activator (PLAU/uPA), vascular endothelial growth factor and endothelium-specific tyrosine kinase 2 [3,30,31,47-50,55,61,65]. The functional interaction analyses in this study further strengthen the importance of Ets-1 in regulating cancer metastasis due to the pathway associations we have observed in WNT, vascular endothelial growth factor, and MMP signaling, as well as ERK5, MAPK, EGF, PDGF, MET and GPCR pathways.

We have previously shown that our Ets-1 ovarian cancer expression model results in changes to cellular metabolism, particularly in the context of glucose utilization and oxygen consumption [32]. Several genes involved in glycolysis, the pentose phosphate pathway and other glycolysis feeder pathways were increased following the overexpression of Ets-1, while key enzymes in the oxidative phosphorylation pathway and the electron transport chain were repressed. Functionally, these cells are unable to grow effectively when glucose is depleted or blocked, suggesting that Ets-1 expression results in a greater reliance on glycolysis for energy generation. In accordance with these results, cellular oxygen consumption was significantly decreased in cancer cells expressing Ets-1, suggesting that electrons are passing through the electron transport chain and generating ROS at a decreased rate. In this study, we were able to repeat these findings in a breast cancer Ets-1 knockdown model, giving more strength to our theory that Ets-1 is integral in regulating cancer cell metabolism. In further support of this role, each of the different pathway analysis approaches used identified enrichments in metabolic pathways, as represented in Figure 3.

Cancer cells display enhanced anabolic nutrient processing, leading to increased rates of protein, nucleic acid and lipid biosynthesis and metabolism. Fatty acid metabolism affects several important pathways involved in cancer progression including cellular signaling and energy processing [66]. The bioinformatics analyses employed here showed enrichments in various fatty acid metabolic pathways, particularly relating to fatty acid desaturation. Fatty acid desaturation by the delta-5 and delta-6 desaturases upregulated by Ets-1 in this study, fatty acid desaturase-1 and −2, result in the production of arachidonic acid, which is associated with breast cancer progression, metastasis and angiogenesis [67-69]. The metabolism of arachidonic acid by lipoxygenases and cyclooxygenases leads to increased ROS production, likely through the stimulation of nicotinamide adenine dinucleotide phosphate-oxidases, leading to the activation of the MAPK pathway, which in turn leads to increased cell proliferation [70]. Though we have not validated this association functionally, the upregulation of fatty acid desaturation by Ets-1 represents another potential mechanism by which this transcription factor enhances cancer progression, and warrants further investigation.

An inevitable consequence of cellular metabolism is the production of ROS, an effect that is amplified in cancer cells due to their altered metabolism, in addition to mitochondrial dysfunction and alterations in antioxidant pathways [16]. As a result of elevated levels of ROS, cancer cells are under high amounts of oxidative stress, leading to enhanced tumor progression. As a signaling molecule, ROS can activate several proliferative signaling pathways including MAPK/ERK, PI3K/Akt and NF-κB [16]. Thus, ROS encourage cancer proliferation, metastasis and angiogenesis, while also inducing apoptosis when left unchecked by cellular antioxidant systems. The pathway analyses in this study have shown common enrichment in metabolic and oxidoreductase pathways in response to Ets-1 overexpression in ovarian cancer cells. These pathways are all important in regulating oxidative stress and cellular redox state, thus it is not surprising that we also observed decreased ROS levels and increased GPX activity in Ets-1 overexpressing cancer cells. GPXs are integral to the control of H_2_O_2_ in cells, and as such have dual pro- and anti-carcinogenic roles depending on the stage of tumor development.

Cancer initiation is facilitated by low levels of GPX enzymes leading to the failure to protect against DNA damage, resulting in genomic instability [71]. In established tumors, loss of GPX activity may promote proliferation and metastasis by allowing the ROS-mediated activation of associated signaling pathways. However, high levels of GPX activity would prevent cellular oxidative damage due to excessive ROS levels leading to improved cancer cell survival through the inhibition of ROS-induced apoptosis. In this study, we have observed the upregulation of GPX1 and 2 protein expression, increased GPX enzyme activity, and decreased intracellular ROS in response to Ets-1 overexpression in ovarian cancer cells. In addition to being relevant to cell apoptosis, the upregulation of GPX1 is also associated with chemotherapeutic resistance in breast cancer [72,73]. GPX2 overexpression is associated with increased cancer cell proliferation through WNT signaling, resistance to apoptosis by reducing ROS, and enhanced growth in breast and intestinal cancers [74-77]. These findings suggest that Ets-1 is important in regulating cellular ROS levels, and thereby regulating cellular redox status and the response to oxidative stress in cancer cells. In further support of this theory, our laboratory has previously shown that Ets-1 transcription can be induced by nuclear factor (erythroid-derived 2)-like 2 (Nrf2) in 2008 ovarian cancer cells [78]. As a master regulator of redox state, Nrf2 initiates key antioxidant pathways to defend against oxidative stress. Notably, this factor is also increased in many types of cancer, and mutations that confer permanent stabilization of Nrf2 are frequently observed [79]. Considering the findings presented in this study, it is possible that Nrf2-mediated induction of Ets-1 is central to the regulation of antioxidant capacity in cancer cells, and thus this may be a promising focus for future studies.

## Conclusions

In this study we have identified some novel pathway associations for Ets-1 transcriptional control, though further study is required to confirm the nature of the gene interactions therein. Interestingly, we have found that several enriched metabolic and oxidative stress pathways are differentially expressed in an ovarian cancer cell model of Ets-1 overexpression. The study of cancer metabolism is a rapidly emerging field in the context of cancer research, though the groundwork for this research was built almost half a century ago [80]. One of the main byproducts of metabolism is ROS, which are produced in excess in cancer cells due to high metabolic rates and mitochondrial dysfunction. We have previously shown that Ets-1 is transcriptionally activated by H_2_O_2_ in ovarian cancer cells via Nrf2 antioxidant response element binding within the Ets-1 promoter [78]. Aggressive cancer cells are often chronically exposed to high levels of oxidative stress, which could explain why Ets-1 is commonly upregulated in these cancers. Therefore, we suggest that increased levels of ROS produced from cancer cells result in the induction of Nrf2 and subsequently Ets-1, which is then involved in a largely undefined transcriptional network that confers metabolic reliance on glycolysis and fat metabolism to fulfill the cancer cell’s high energy needs. As ROS have such integral roles in cancer progression, an intimate understanding of the underlying mechanisms that achieve elevated levels of ROS will open the door to novel molecular avenues for drug development.

## Abbreviations

2-DG: 2-Deoxy-D-glucose; AFU: Arbitrary fluorescent units; EGF: Epidermal growth factor; ERK: Extracellular-signal-regulated kinase; Ets: E-26; FDR: False discovery rate; FI: Function interaction; GPCR: G protein-coupled receptor; GPX: Glutathione peroxidases; GSEA: Gene set enrichment analysis; IC50: Dose at which 50% of cells stop proliferating; MAPK: Mitogen-activated protein kinase; MMP: Matrix metalloproteinase; Nrf2: Nuclear factor (erythroid-derived 2)-like 2; PDGF: Platelet-derived growth factor; qRT-PCR: Quantitative reverse-transcription polymerase chain reaction; ROS: Reactive oxygen species; WNT: Wingless-type.

## Competing interests

The authors declare that they have no competing interests.

## Authors’ contributions

MV carried out bioinformatic analyses, all functional assays, and drafted the manuscript. CV participated in the gene ontology analysis and aided in statistical analysis. GS participated in the study design and coordination, and revised the manuscript. All authors read and approved the final manuscript.

## Supplementary Material

Additional file 1 Real time qRT-PCR primer sequences used for gene expression analysis. Click here for file

Additional file 2 List of enriched genes from each outlined module from Reactome FI global interaction network. Click here for file

Additional file 3 List of enriched genes from the mitochondria and fatty acid metabolism pathways from GSEA analysis. Click here for file
