# Supplementary figures and images for: Ets-1 global gene expression profile reveals associations with metabolism and oxidative stress in ovarian and breast cancers
Source: Cancer Metab. 2013 Jul 25;1:17. doi: 10.1186/2049-3002-1-17 (PMC4178218; doi:10.1186/2049-3002-1-17)

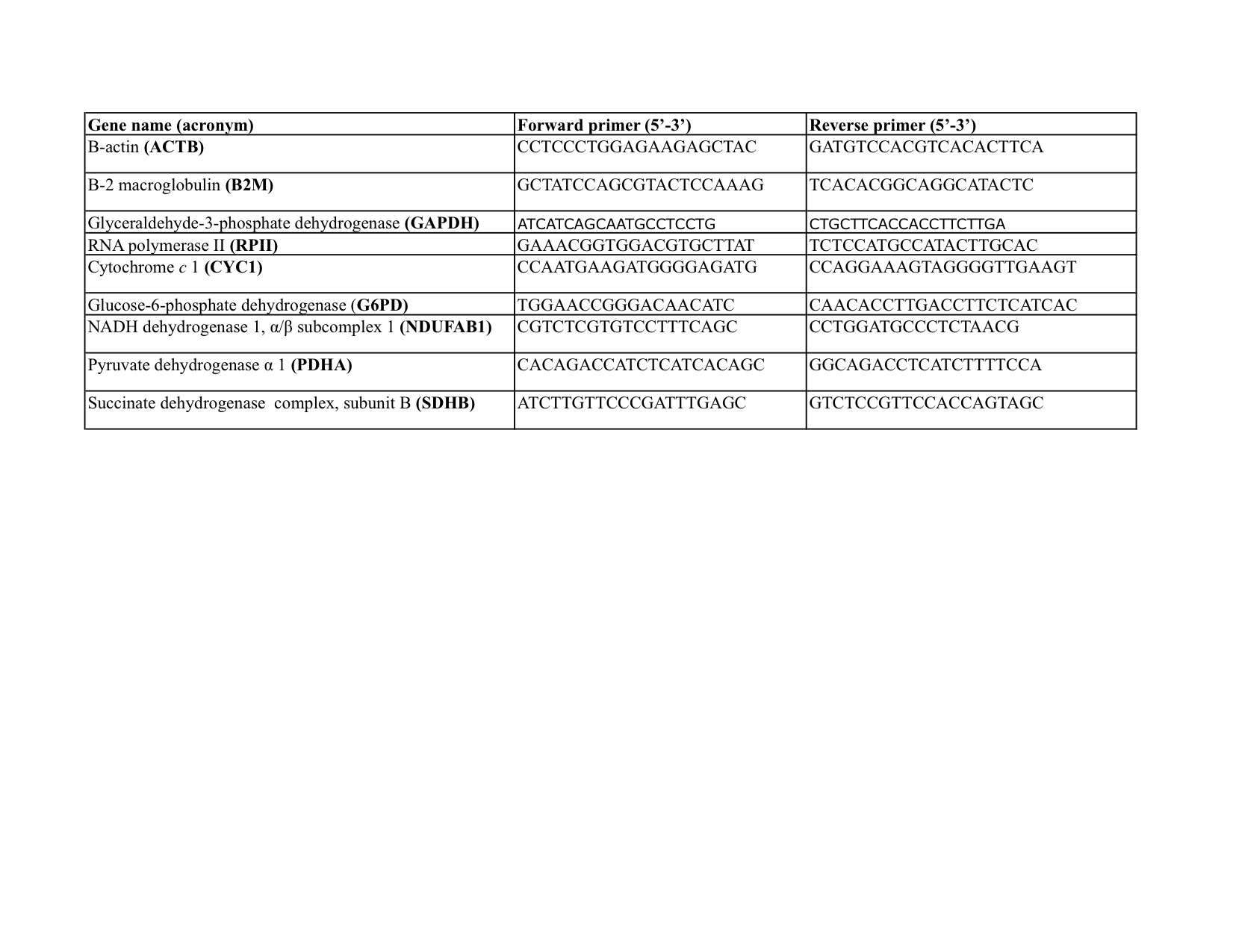

Supplement: Additional file 1 — Real time qRT-PCR primer sequences used for gene expression analysis. [file 2049-3002-1-17-S1.jpeg]
